# Challenges of training and delivery of pediatric surgical services in developing economies: a perspective from Pakistan

**DOI:** 10.1186/s12887-019-1512-9

**Published:** 2019-05-16

**Authors:** Amir Humza Sohail, Muhammad Hassaan Arif Maan, Mohammed Sachal, Muhammad Soban

**Affiliations:** 1 Department of Surgery, Howard University Hospital, Washington, DC USA; 2 Medical College, The Aga Khan University, Stadium Road, Karachi, Pakistan; 3 King Edward Medical University, Lahore, Pakistan; 4 The Aga Khan University, Karachi, Pakistan

**Keywords:** Pediatric surgery, Pediatric workforce, Specialized surgical care, Low- and middle-income countries

## Abstract

**Background:**

As the pediatric population requiring health services rises globally, developing countries are struggling to cater to the growing burden of non-communicable diseases - particularly those requiring specialized surgical care.

**Main body:**

Despite the literature supporting specialized pediatric surgical care, the developing world is far from meeting the American Pediatric Surgical Association (APSA) Manpower taskforce recommendation of at least 1 qualified pediatric surgeon per 100,000 patients (0–15 years-old). In Pakistan, there is an unmet surgical need in the pediatric population due to a multitude of short shortcomings, notably in quality and quantity of the training programs on offer, and urgent short- and long-term steps are needed to improve this dire situation.

**Conclusion:**

It is crucial for the global surgical community to take steps, especially with regards to pediatric surgical training, to ensure delivery of accessible and quality surgical care to the world’s children.

## Background

The pediatric population requiring health services is rising globally. [1] Interestingly, while tremendous advances have been made in the formulation of evidence-based strategies and policies geared towards prevention and management of communicable diseases in this population group, non-communicable diseases – particularly those requiring specialized surgical care – are often neglected, especially in developing countries. Furthermore, improved outcomes have been associated with care provided by pediatric surgical subspecialists with advanced training for children requiring surgery than that delivered by other healthcare professionals. [2–12] Thus, it is crucial to ensure the provision of relevant infrastructure and pediatric surgery training opportunities to cater to the ever-growing burden of surgical conditions in the pediatric population.

## Main text

The American Pediatric Surgical Association (APSA) Manpower taskforce recommends that the number of qualified pediatric surgeons in a population should be at least 2 per million (or 1 per 100,000 patients between 0 and 15 years of age). [13] Even though only a handful of countries (e.g. the US, Finland, Canada, Australia and Switzerland) meet the above-mentioned standards, the growth rate of pediatric surgical graduates’ numbers in the western world in recent years is higher than that previously forecasted, which provides some reassurance. [13, 14] However, data from developing countries are less encouraging. For instance, the reported numbers of pediatric surgeons (per hundred thousand population) in Asian countries (e.g. Bangladesh, 0·30; India, 0·28; Pakistan, 0·26; Indonesia, 0·03; and Malaysia, 0·22) is suboptimal. [14] This shortage of pediatric surgeons, in conjunction with other hurdles to quality healthcare in resource-limited settings, has dire consequences for population health. For example, according to an estimate in 2015, Nepal has more than 700,000 children with unmet needs for surgical care. [15] Butler et al., while focusing on four low- and middle-income countries (LMICs) (Rwanda, Sierra Leone, Nepal and Uganda) found that 62% of children (3.4 million children) in need of surgical intervention had not received the required care. [16] This highlights the need to bridge gaps in provision of specialized pediatric surgical care in LMICs. In view of the growing global pediatric population and the mounting needs for surgical care that this entails, the current sub-optimally planned approach to pediatric surgery will inevitably lead to crises in health service delivery mechanisms, particularly in LMICs.

In Pakistan, reasons for the mismatch between the number of graduating pediatric surgeons and the growing population needs are manifold. [17] Pediatric surgery training involves post-graduate fellowships at major institutions under the aegis of College of Physicians and Surgeons, Pakistan (CPSP). [17] Variations in training programs offered by different institutions, despite the presence of a CPSP standardized curriculum, need to be addressed. Furthermore, we propose that greater exposure to pediatric surgery in formative medical training may result in greater motivation to pursue a career in pediatric surgery among young surgical/medical graduates. [17, 18] To tackle these issues, a multi-pronged strategy is required. Incorporation of some pediatric surgical care training into general surgical training programs will not only instill the required skills and confidence in general surgeons to handle pediatric patients, especially in rural areas without access to pediatric surgery specialists, but may also spawn their interest in this field as a potential fellowship option. Reforms to simplify the lengthy CPSP accreditation process could also pave the way for setting up of new fellowship programs.

## Conclusion

Challenges, particularly shortage of training opportunities and administrative hurdles in developing pediatric surgery training programs, hamper delivery of accessible and quality surgical care to the world’s children. The global surgical community and individuals in leadership roles, especially in developing economies, must recognize the need to address the current pitfalls and the emerging challenges in pediatric surgery.

## Abbreviations

APSA: American Pediatric Surgical Association
CPSP: College of Physicians and Surgeons, Pakistan
LMIC: Low- and middle-income countries
